# Naphthoquinones from *Handroanthus impetiginosus* promote skin wound healing through Sirt3 regulation

**DOI:** 10.22038/ijbms.2020.43706.10275

**Published:** 2020-09

**Authors:** Fayyaz Ahmad, Shaheen Bibi, Mincheol Kang, Mariam Anees, Muhammad Ansar, Muhammad Rizwan Alam, Sun Yeou Kim, Hussain Mustatab Wahedi

**Affiliations:** 1 Department of Biochemistry, Faculty of Biological Sciences, Quaid-i-Azam University, 45320 Islamabad, Pakistan; 2 College of Pharmacy, Gachon University, 191 Hambakmaero, Incheon, South Korea; 3 Department of Biological Sciences, National University of Medical Sciences, C/O Military Hospital, Mall Road Rawalpindi, Pakistan

**Keywords:** Beta-Lapachone, Dermatology, Inflammation, Regeneration, Tabebuia

## Abstract

**Objective(s):**

Lapachone is a natural naphthoquinone-derived compound found in *Tabebuia avellanedae*. It is well-known for its analgesic, anti-inflammatory, anti-microbial, diuretic, and anti-cancerous effects. However, the wound-healing effects of this compound are not known yet. The aim of this study was to investigate the wound healing activity of naphthoquinones (α-lapachone and β-lapachone) from *Handroanthus impetiginosus*.

**Materials and Methods:**

Expression of Sirt3, migration-related proteins (Rac1, Cdc42, α-Pak) and angiogenesis-related protein of vascular endothelial growth factor (VEGF) was monitored using western blot analysis. Blood vessel formation and tissue development were monitored by angiogenesis assay and hematoxylin & eosin (H & E) staining, respectively on mouse skin tissue samples. Both α-lapachone and β-lapachone increased Sirt3 expression *in vivo*, but only β-lapachone increased Sirt3 expression *in vitro. *

**Results:**

Both the compounds accelerated wound healing in cultured skin cells as well as mouse skin; however, β-lapachone was more effective at lower concentrations. Both of the compounds increased the expression of migration-related proteins both *in vitro *and *in vivo*. Similarly, α-lapachone and β-lapachone increased VEGF expression, tissue development and blood vessel formation in mouse skin.

**Conclusion:**

These findings indicated that α-lapachone and β-lapachone are novel Sirt3 activators, and Sirt3 has a role in wound healing. Thus, Sirt3 and its regulators come out as a novel target and potential drug candidates, respectively in the important field of cutaneous wound healing.

## Introduction

Cutaneous wound healing is a very complex biological process, which is regulated by several factors and is divided into four overlapping phases: hemostasis, inflammation, proliferation and tissue remodeling (1). In the hemostasis phase, platelets play a key role to form the clot. They promote clotting by releasing chemical signals to seal the injury. By preventing further bleeding, platelets stick together to activate fibrin in the blood vessels to form the clot (2). The inflammation consists of two phases, early inflammatory phase, and late inflammatory phase. In the early inflammatory phase, the white blood cells engulf the debris by a process called phagocytosis (3). In the late inflammatory phase (48-72 hr after injury), the process of phagocytosis is initiated by macrophages at the wound sites (4). Macrophages are very important for the late stage of the inflammatory response, because of their potential to provide tissue growth factors, especially transforming growth factor beta (TGF-beta) and other mediators (TGF-alpha, fibroblast growth factor, and collagenase) that are required for the activation of fibroblast, endothelial cells, and keratinocytes (5). In the proliferation phase, major events like fibroblast migration, collagen synthesis, angiogenesis and granulation tissue formation take place (6). Rho GTPase family members Rac1 and Cdc42 play a pivotal role in cell migration along with alpha pak (7). The phenomenon of wound healing is linked to the amendments in the cytoskeletal entities, which are brought about by the members of the Rho family of GTPases (8). The remodeling phase starts with the synthesis of extracellular matrix and granulation tissue development (9).

*Handroanthus impetiginosus* is an ornamental tree, also called pink lapacho, which belongs to the Bignoniaceae family and shows high pink color during spring. The stem of this tree possesses anti-inflammatory, antimicrobial, diuretic, and anti-carcinogenic properties, whereas its leaves are used for the antiseptic and astringent activity. 

Lapachone is a natural compound that is obtained from the bark of *Tabebuia avellanedae*, commonly known as the Lapacho tree. It is a 0-naphthoquinone derived compound existing in two related isoforms called α-lapachone and β-lapachone (Figure S1 A and B). It has been used for different therapeutic purposes in Brazil such as analgesic, anti-inflammatory, antimicrobial, diuretic and anti-cancer (10, 11). Similarly, juglone, a naphthoquinone, found in walnuts possesses anti-cancer, anti-proliferative (12, 13) anti-inflammatory, antimicrobial and anti-aging effects (14-16). It has been previously reported that juglone upregulates Sirt1 in normal and UVB-irradiated skin cells (17). Moreover, it is also involved in invasion and migration of lung cells (18).

Sirtuins (Sirt) are proteins of a much-conserved family of ADP-ribosyltransferases and NAD+ dependent deacetylases. They play a regulatory role in various life processes such as aging, cancer and inflammatory response (19). Sirt3 has been shown as a key player in skin maintenance via oxidative stress-induced keratinocytes differentiation. It is an important process for skin regeneration and maintenance and is significant in skin disease (20). Administration of resveratrol or MC2562 results in the activation of Sirt-1, Sirt-2, and Sirt-3, which accelerates wound repair in a mouse model through increased keratinocytes proliferation (21). This study aimed to explore the wound healing potential of naphthoquinones from *H. impetiginosus* through Sirt3 activation in human skin cells and mouse models. 

## Materials and Methods

***Cell culture***

HaCaT cells were purchased from the Korean cell line bank. The human epidermal cells (HaCaT) were cultured in DMEM (Dulbecco’s Modified Eagle’s Medium, Thermo-scientific, Waltham, MA) with 10% fetal bovine serum (FBS) and 1 % penicillin-streptomycin and were incubated in 5% CO_2_ at 37 ^°^C with 95% humidity.

***Treatment with α-lapachone and β-lapachone***

HaCaT cells were cultured in 6-well plates (1.2 x 10^5^ cells/well). Once cultures reached 80 % confluency, they were rinsed with phosphate-buffered saline (PBS). For treatment with α-lapachone and β-lapachone (Sigma Aldrich Co Ltd, MI, USA), the stock solution was diluted in the appropriate volume of fresh serum-free media to make dilutions of 1 µM, 5 µM, 10 µM, and 25 µM. After the treatment, cultures were placed in a humidified atmosphere at 37 ^°^C with 5% CO_2_ until harvested for immunoblotting. 

***Cell migration assay***

HaCaT cells were seeded in 96-well plates. Monolayer cultured cells were subjected to the scratch wound with the wound maker tool (Essen Bioscience, Ann Arbor, MI), and media was removed by aspiration. Cells were then washed twice in PBS and incubated in the presence or absence of α and β-lapachone in serum-free media for 24 hr. The cultures were monitored every 4 hr with the IncuCyte Zoom (Essen Biosystem) imaging system.

***Full-thickness wounds and quantification of healing***

Three to four-week-old albino mice were divided into five different groups with 10 mice in each group; i.e., Vehicle, 0.05% α-lapachone, 0.1% α-lapachone, 0.05% β-lapachone and 0.1% β-lapachone. A 4 mm biopsy punch was used to create two wounds in the dorsal posterior region of all mice after hair removal. Different concentrations of lapachone solutions (0.05% α-lapachone, 0.1% α-lapachone, 0.05% β-lapachone and 0.1% β-lapachone) were prepared in a solvent containing distilled water, ethanol and propanediol (5:3:2), and 200 µl/mouse was topically applied to the wounded areas once a day for 10 consecutive days. The vehicle group was treated only with the solvent. Wounded mice were digitally photographed individually after treatment from day 0 to day 10. Mice were sacrificed on day 3, 7 and 10 post-wounding to collect skin samples. Skin samples were subjected to tissue lysis for protein isolation using lysis buffer mentioned below. 

***Protein extraction and quantification***

Mice skin tissues collected during wound closure at days 3, 7 and 10 were treated with 400 µl lysis buffer (10 mM Tris-HCl [PH: 8], 1 mM EDTA, 140 mM NaCl, 1% Triton X-100, 0.1% SDS and 1% of protease inhibitor prepared in dH_2_O). 250 µl buffer was added in 100 mg skin tissue present in an Eppendorf tube. The skin was meshed and grinded by using scissors and blades. In case of cells, the cell pellet was mixed with lysis buffer and incubated on ice for 30 min. The samples were centrifuged at 14,000 rpm for 15 min at 4 ^°^C. The upper layer containing proteins was picked up and stored at -20 ^°^C for Western blot analysis. The Bradford assay was used to determine the total protein concentration of a sample using bovine serum albumin (BSA) as standard. Total of eight serial dilutions of BSA (Cat No: 39222.01, SERVA Electrophoresis, Heidelberg Germany) standard were prepared at concentrations of 0 µg/µl, 0.2 µg/µl, 0.4 µg/µl, 0.6 µg/µl, 0.8 µg/µl, 1.0 µg/µl, 1.2 µg/µl and 1.4 µg/µl in 1.5 ml Eppendorf tubes. 3 µl of each BSA standard and skin/cell protein samples were loaded in a 96-well plate in triplicate, and 200 µl of 5X Bradford reagent (Cat No: 39222.01, SERVA Electrophoresis, Heidelberg Germany) was added to each well. The 96-well plate was incubated at room temperature for 10 min and absorbance of each sample was measured at 595 nm using a UV-visible spectrophotometer.

***Western blotting***

For expression analysis of individual proteins of interest, protein samples (30 μg) were subjected to SDS-PAGE (poly acrylamide gel electrophoresis), which were then transferred onto the nitrocellulose membranes. The membrane was blocked with fat-free skimmed milk blocking solution for 1 hr on shaker, followed by the incubation with the primary antibodies (Sirt3, VEGF, α-Pak, Cdc42, Rac1, and Rho-A) at 1: 1000 dilutions for overnight at 4^ °^C. After that, the membrane was again incubated with secondary antibodies (Mouse anti-rabbit IgG and m-IgGk) conjugated with horseradish peroxidase in 1:5000 dilution at room temperature for 2 hr. Finally, the membrane was incubated with the substrate (luminal) and peroxide solution (hydrogen peroxide) for 5 min. The membrane was put in a gel documentation system (Proteinsimple, CA, USA) to observe the protein bands expression and pictures were captured.

***Histological analysis***

Mice were sacrificed at days 3, 7 and 10 post-wounding. Skin samples were collected containing the wound areas for histological study and placed in 10% formalin. Samples were embedded in paraffin wax and cut into 5-µm-thick sections for hematoxylin and eosin (H & E) and Masson’s trichrome staining.

***Angiogenesis assay or blood vessel formation assay***

Sponges of equal size (mm of diameter and 2.75 mm thick) were hydrated by keeping them in 0.9% saline solution overnight. Two sponges, oppositely placed to each other, were inserted in the abdomen of each mouse by making vertical incisions. Two to three sutures were placed to close the incision. BD syringes of 1 ml were used to inject lapachone solutions in sponges. Sponges were subsequently harvested and processed for histological processing.

***Statistical analysis***

Differences between groups were determined using a one-way analysis of variance (ANOVA). *P*-values of <0.05, <0.01, and <0.001 were considered statistically significant. Results are presented as the mean with the standard error of the mean (SEM).

## Results

***Effects of α-lapachone and β-lapachone on cell migration and wound healing***

**Figure 1:**
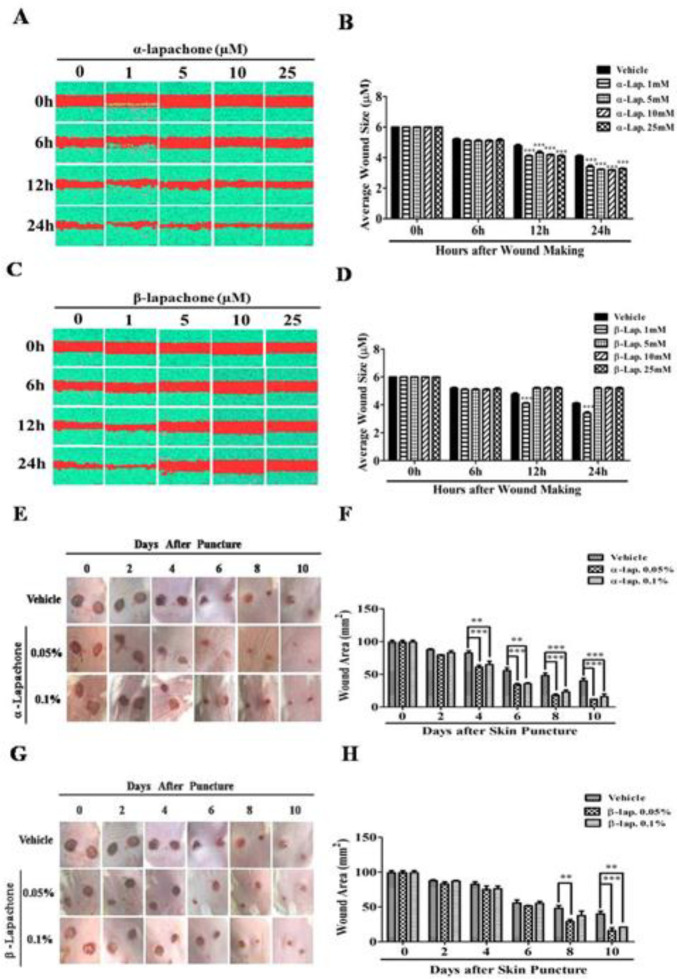
Effects of α-lapachone and β-lapachone on cell migration and wound healing. (A) Effects of α-lapachone on the migration rate of epidermal keratinocytes. (B) Average wound size in α-lapachone treated cells plotted as a bar graph. (C) Effects of β-lapachone on the migration rate of epidermal keratinocytes. (D) Average wound size in β-lapachone treated cells plotted as a bar graph. Values are means±SEM. ∗∗∗*P*<0.001 VS vehicle group. (E) Effects of α-lapachone on wound healing in mice. (F) Graphical representation of the effects of α-lapachone on wound healing in mice. (G) Effects of β-lapachone on wound healing in mice. (H) Graphical representation of the effects of β-lapachone on wound healing in mice. Values are means±SEM (n = 5/group). ∗∗*P*<0.01, ∗∗∗*P*<0.001 VS vehicle at different time points

The migration rate of epidermal keratinocytes after treatment with α-lapachone was not different from untreated cells for up to 6 hr. However, treatment with 1 µM α-lapachone, 5 µM α-lapachone, 10 µM α-lapachone, and 25 µM α-lapachone accelerated the migration rate as compared to the vehicle from 12 hr onwards (Figure 1A). The average wound size was significantly reduced in epidermal keratinocytes after treatment with 1 µM α-lapachone, 5 µM α-lapachone, 10 µM α-lapachone and 25 µM α-lapachone (Figure 1B).

The migration rate of epidermal keratinocytes after treatment with different concentrations of β-lapachone was the same for up to 6 hr. However, treatment with 1 µM β-lapachone enhanced the migration rate from 12 hr onwards as compared to the vehicle (Figure 1C). The average wound size in epidermal keratinocytes was also determined after treatment with different concentrations of β-lapachone (Figure 1D). The average wound size was significantly reduced in epidermal keratinocytes after treatment with 1 µM β-lapachone as compared to the vehicle (Figure 1D).

The activity of different concentrations of α-lapachone on wound healing was analyzed at different days after wound creation. Slower wound healing occurred in hairless mice treated with vehicle as compared to mice treated with α and β-lapachone solutions (Figure 1E). A significant increase in the rate of wound healing was observed in α-lapachone as compared to the vehicle because the wound area was significantly reduced at day 10 in α-lapachone treated mice (Figure 1E). This difference in wound area was significant from day 4 onwards in both 0.05% and 0.1% α-lapachone treated mice (Figure 1F). The activity of different concentrations of β-lapachone on wound healing was analyzed at different days after wound creation. A clear difference of wound closure was observed from day 6 onwards, especially in mice treated with 0.05% β-lapachone (Figure 1G). The wound area was significantly decreased at day 10 in mice treated with 0.05% and 0.1 % β-lapachone as compared to vehicle (Figure 1H).

***Effects of α-lapachone and β-lapachone on expression of Sirt3 in epidermal keratinocytes and in mouse skin***

**Figure 2:**
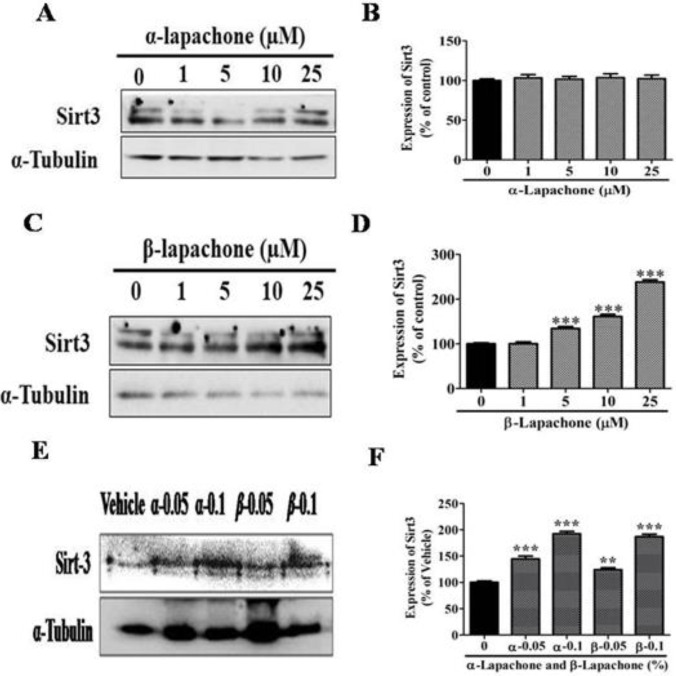
Effects of α-lapachone and β-lapachone on expression of sirtuin3 in epidermal keratinocytes and in mouse skin

The expression of Sirt3 was analyzed in epidermal keratinocytes after treatment with different concentrations of α-lapachone by Western blot analysis (Figure 2A). The expression of Sirt3 was not increased significantly in the skin keratinocytes by any concentration of α-lapachone (Figure 2B).

However, expression of Sirt3 notably increased followed by treatment with different concentrations of β-lapachone in a dose-dependent manner as compared to vehicle-treated cells (Figure 2C). This increase in Sirt3 expression was more significant, especially at higher concentrations i.e. 10 and 25 µM (Figure 2D). The mice’s skin was treated with different concentrations of α and β-lapachone to investigate the expression of Sirt3. The expression level of Sirt3 was significantly upregulated after treatment with both α and β-lapachone at all concentrations (Figure 2E). But, the increase in Sirt3 protein level was more significant at 0.1% concentrations of both α and β-lapachone (Figure 2F).

***Effects of α-Lapachone and β-Lapachone on migration-related proteins in skin cells***

**Figure 3:**
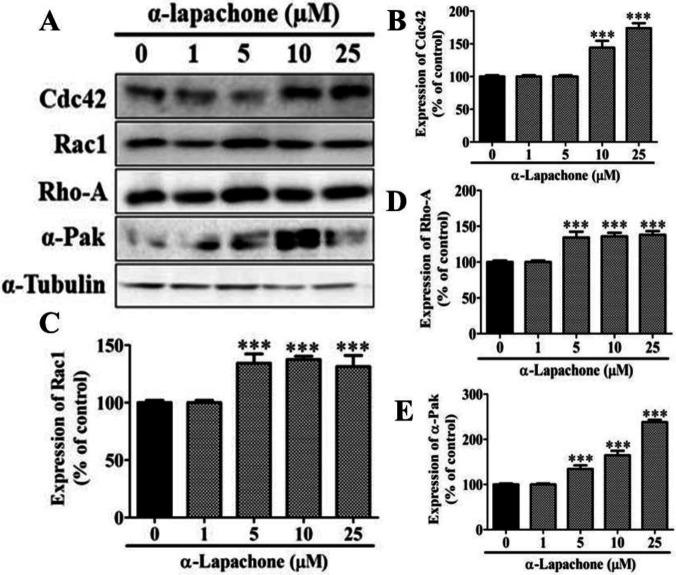
Effects of α-lapachone on migration-related proteins in skin cells

The effect of different concentrations of α-lapachone on the expression of migration-related proteins in epidermal keratinocytes was determined by Western blot analysis. The expression of migration-related proteins (Cdc42, Rac1, Rho-A, and α-Pak) was increased with different concentrations of α-lapachone in a dose-dependent manner (Figure 3A). The expression of Cdc42 was significantly elevated with 10 and 25 µM α-lapachone as compared to the untreated cells (Figure 3B). The expression of Rac1, on the other hand, was significantly increased upon treatment with 5, 10 and 25 µM α-lapachone in a dose-dependent manner (Figure 3C). A similar expression pattern was observed for RhoA and α-Pak (Figures 3D-E).

**Figure 4:**
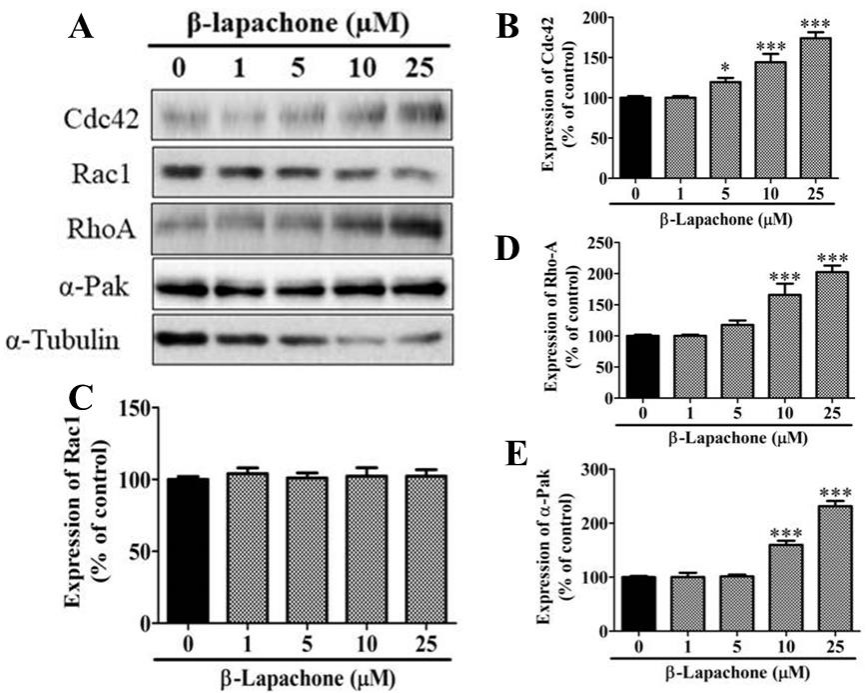
Effects of β-lapachone on migration-related proteins in skin cells

The expression of most of the migration-related proteins (Cdc42, Rho-A, and α-Pak) was increased with different concentrations of β-lapachone in a dose-dependent manner (Figure 4A). However, Rac1 did not show any significant rise at any concentration (Figure 4C). The expression of Cdc42 was significantly upregulated with 5, 10 and 25 µM β-lapachone as compared to untreated cells (Figure 4B). A similar expression pattern was observed for RhoA and α-Pak (Figures 4D-E).

***Effects of α-lapachone and β-lapachone on tissue morphology and angiogenesis***

**Figure 5:**
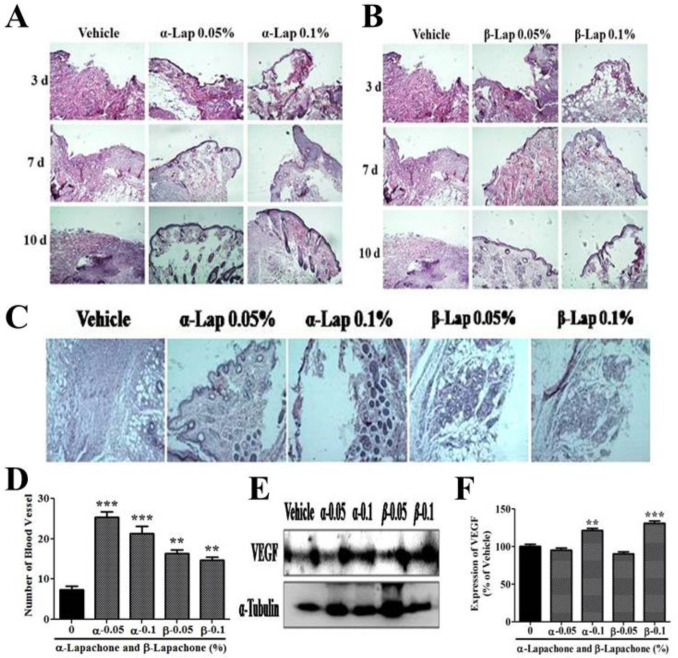
Effects of α-lapachone and β-lapachone on tissue morphology and angiogenesis

The mice’s skin tissue was treated with α-lapachone to evaluate the neuroepithelium and granulation tissue formation. There were faster dermis and epidermis formation in mice treated with α-lapachone as compared to the vehicle (Figure 5A). The formation of dermis and epidermis in mice treated with α-lapachone was better at day 3 and day 7 as compared to the vehicle group. Moreover, dermal and epidermal layers were completely formed at day 10 in mice treated with 0.05% α-lapachone and 0.1% α-lapachone while not completely formed in the vehicle group (Figure 5A).

The dermis and epidermis were not clearly formed/ observed in mice treated with β-lapachone at day 3 but clearly formed/observed in mice treated with β-lapachone 0.05% at day 7 and day 10 as contrary to the mice treated with vehicle. However, β-lapachone 0.1% did not show any significant effect on tissue development (Figure 5B).

There was a high number of blood vessels in mice skin treated with 0.05 % α-lapachone and 0.1 % α-lapachone as compared to the vehicle (Figure 5D). The higher numbers of blood vessels were also observed in 0.05% β-lapachone as compared to the vehicle and 0.1% β-lapachone. The overall result demonstrated a higher number of vessel formation in both α-lapachone and β-lapachone treated mice as compared to vehicle-treated mice, which had a low number of blood vessels. In order to further confirm this data, expression of vascular endothelial growth factor (VEGF) was also analyzed in mouse skin after treatment with different concentrations of α and β-lapachone (Figure 5E). A high expression level of VEGF was observed in 0.1% α-lapachone and 0.1% β-lapachone as compared to the vehicle group (Figure 5E). Whereas, the expression of VEGF in 0.05% α-lapachone and 0.05% β-lapachone group was not affected (Figure 5F).

## Discussion

The effect of both forms of lapachone on the wound healing rate was very significant in cell lines as well as in the animal model. The migration rate of cultured cells and the healing of full-thickness wounds in mice were significantly increased. These findings can be attributed directly or indirectly to the upregulation of Sirt3 as it was also obvious both i*n vitro* and i*n vivo*. Major events like cell migration, tissue development, and angiogenesis are all ameliorated as evident from Western blot analysis, histological analysis and angiogenesis assays, which indicates that lapachone enhances almost all major phases of the wound healing.

Alpha lapachone did not increase the expression of Sirt3 *in vitro*. *In vitro* data showed that the expression of Sirt3 was increased after treatment with β-lapachone. The expression of Sirt3 was increased after the treatment of different concentrations of both α and β-lapachone *in vivo*. This may be because of different conditions for *in vivo* and *in vitro* experiments. Moreover, α-lapachone may increase the expression of some sirtuins, which can be confirmed in future studies. Experiments performed using skin cell lines and mouse models indicated the wound healing effects of both forms of lapachone. Cultured skin keratinocytes indicated that treatment with α-lapchone increased the rate of cell migration. This increase was dose-dependent and was very clear and significant from 12 hr onwards. Similar results were found for β-lapachone with the difference that β-lapachone was more effective at lower concentrations. These results are very clear and meaningful for the process of wound healing. During the proliferation phase of wound healing, one of the major events is the migration of the epidermal keratinocytes towards the wound area. Dose-dependent migration of cells shows a clear role of alpha lapachone in migration. However, β-lapachone may require further investigation since it may have a different optimum concentration, which is why its effect is not increasing with dose. β-lapachone may have low effective concentration like juglone as we previously reported (22). Alpha lapachone, as suspected, increased the expression of migration-related proteins like Rac1, Alpha Pak, Cdc42 and RhoA. All these proteins increased with an increasing dose of the α-lapachone. Cell migration as an energy-requiring process involves a lot of changes at the subcellular level. One of the most important events during cell migration is the cytoskeletal rearrangements (7). Rac1 and Cdc42 make a complex that interacts with α-Pak. These three proteins in concert with Rho GTPase, and Rho-A induce necessary changes in the cytoskeleton to create polarity in the cell. This polar cell then starts migrating towards the anterior end (7, 23). 

Wound healing effects of α and β-lapachone were tested in the animal model to confirm the *in vitro* results. As expected, α-lapachone was effective in the mouse skin in a dose-dependent manner just like in the cell culture. Previous findings also showed that β-lapachone promotes the proliferation of different cells (fibroblast, keratinocytes and endothelial cells) at low concentrations (0.1 µM) and thus enhancing wound healing activity (10). 

Both concentrations of α-lapachone (0.05% and 0.1%) were equally effective and increased wound healing rate from day 4 onwards. However, β-lapachone was more effective at lower concentrations after day 6 post-injury. These findings suggest that α-lapachone is more effective over a wide range of concentrations, while β-lapachone shows the effect at specific concentrations. A similar pattern of the two compounds was visible in the tissue development rate as well. The dermis and epidermis were restored rapidly in mice skin treated with different concentrations of α and β-lapachone as compared to the vehicle. The present study also reported that the number of the blood vessel was high in lapachone-treated skin samples, confirming the idea that both forms of lapachone are effective for the increased rate of wound healing.

(A) Effects of α-lapachone on the expression of Sirt3 in epidermal keratinocytes. (B) Graphical representation of the expression pattern of Sirt3. (C) Effects of β-lapachone on the expression of Sirt3 in epidermal keratinocytes. (D) Graphical representation of the expression pattern of Sirt3. (E) Effects of α and β-lapachone on the expression of Sirt3 proteins in mouse skin. (F) Graphical representation of the expression pattern of Sirt3 protein in mouse skin. Values are means±SEM. ∗∗*P*< 0.01 and ∗∗∗*P*<0.001 VS vehicle/control group

(A) Effects of α-lapachone on the expression of migration-related proteins in epidermal keratinocytes. (B) Graphical representation of the expression pattern of Cdc42. (C) Graphical representation of the expression pattern of Rac1. (D) Graphical representation of the expression pattern of Rho-A. (E) Graphical representation of the expression pattern of α-Pak. Values are means±SEM. ∗∗∗*P*<0.001 VS vehicle/control group

(A) Effects of β-lapachone on the expression of migration-related proteins in epidermal keratinocytes. (B) Graphical representation of the expression pattern of Cdc42. (C) Graphical representation of the expression pattern of Rac1. (D) Graphical representation of the expression pattern of Rho-A. (E) Graphical representation of the expression pattern of α-Pak. Values are means±SEM. ∗∗∗*P*<0.001 VS vehicle/control group

(A) Effects of α-lapachone on tissue development in wounded mouse skin. (B) Effects of β-lapachone on tissue development in wounded mouse skin. (C) Effects of α and β-lapachone on blood vessel formation. (D) Graphical representation of the effects of α and β-lapachone on blood vessel formation. (E) Expression of VEGF after treatment of α and β-lapachone. (F) Graphical representation of the expression pattern of VEGF (n=3). Values are means±SEM. ∗∗*P*<0.01 and ∗∗∗*P*<0.001 VS vehicle group. VEGF: vascular endothelial growth factor

**Figure 6:**
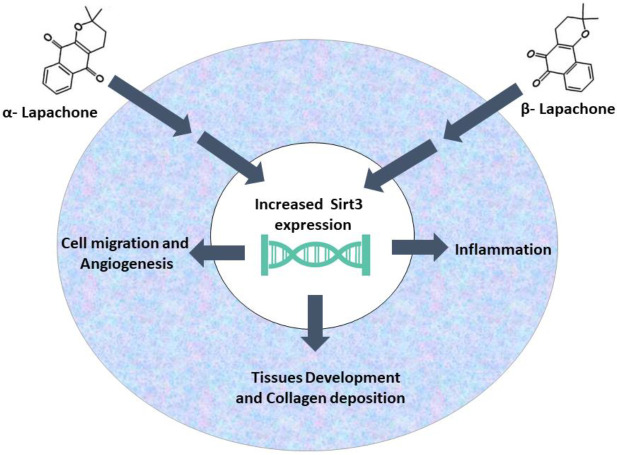
Summary of the wound healing effects of α-lapachone and β-ladapachone

## Conclusion

This data indicates that α and β-lapachone increase the rate of wound healing by ameliorating the rate of cell migration during the proliferation phase by increasing the expression of cell migration-related proteins. These compounds also elevate the expression of Sirt3 indicating the involvement of Sirt family proteins in the process of cutaneous wound healing and skin regeneration. The two compounds being structurally related have similar outcomes but show different effective and optimum concentrations as α-lapachone shows the effect at almost all concentrations and β-lapachone yielded effect at low concentrations (Figure 6).
